# Assessing risk of bias in toxicological studies in the era of artificial intelligence

**DOI:** 10.1007/s00204-025-03978-5

**Published:** 2025-07-04

**Authors:** Thomas Hartung, Sebastian Hoffmann, Paul Whaley

**Affiliations:** 1 CAAT-Europe, University of Konstanz, Constance, Germany; 2 Bloomberg School of Public Health and Whiting School of Engineering, Center for Alternatives to Animal Testing (CAAT), Johns Hopkins University, Baltimore, MD USA; 3 Doerenkamp-Zbinden Chair for Evidence-Based Toxicology, Baltimore, MD USA; 4 Evidence-Based Toxicology Collaboration and Johns Hopkins University Bloomberg School of Public Health, Baltimore, MD USA; 5 Seh Consulting + Services, Paderborn, Germany; 6 Lancaster Environment Centre, Lancaster University, Lancaster, UK

**Keywords:** Risk of bias, Artificial intelligence, Toxicology, Evidence-based toxicology, Systematic review, SYRCLE, OHAT, ToxRTool, AI bias, Regulatory toxicology

## Abstract

Risk of bias is a critical factor influencing the reliability and validity of toxicological studies, impacting evidence synthesis and decision-making in regulatory and public health contexts. The traditional approaches for assessing risk of bias are often subjective and time-consuming. Recent advancements in artificial intelligence (AI) offer promising solutions for automating and enhancing bias detection and evaluation. This article reviews key types of biases—such as selection, performance, detection, attrition, and reporting biases—in in vivo, in vitro, and in silico studies. It further discusses specialized tools, including the SYRCLE and OHAT frameworks, designed to address such biases. The integration of AI-based tools into risk of bias assessments can significantly improve the efficiency, consistency, and accuracy of evaluations. However, AI models are themselves susceptible to algorithmic and data biases, necessitating robust validation and transparency in their development. The article highlights the need for standardized, AI-enabled risk of bias assessment methodologies, training, and policy implementation to mitigate biases in AI-driven analyses. The strategies for leveraging AI to screen studies, detect anomalies, and support systematic reviews are explored. By adopting these advanced methodologies, toxicologists and regulators can enhance the quality and reliability of toxicological evidence, promoting evidence-based practices and ensuring more informed decision-making. The way forward includes fostering interdisciplinary collaboration, developing bias-resilient AI models, and creating a research culture that actively addresses bias through transparent and rigorous practices.

*“Quality is not an act, it is a habit.”*

Aristotle (384-322 BC)

*“You can't fake quality any more than you can fake a good meal.”*

William S. Burroughs (1914–1997)

## Introduction

Internal validity, i.e., the degree to which a study’s results are accurate and can be used to establish a cause-and-effect relationship (Patino and Ferreira 2018), a critical aspect of study quality, refers to the biases (i.e., systematic errors or deviations from the truth in results or inferences) that can arise due to flaws in the design, conduct, analysis, or reporting of a study. Internal validity is the construct, risk of bias assessment is the method we use for assessing it (Frampton et al. 2022). These biases can lead to an overestimation or underestimation of the true effect of an intervention or exposure, and thus to incorrect conclusions about its safety or toxicity. Assessing potential for bias is a fundamental step in the systematic review process (Hoffmann et al. 2017), as studies with high risk of bias can distort the overall evidence synthesis and lead to inappropriate recommendations or decisions.

Bias is distinct from other issues affecting research quality, specifically:

1. Imprecision refers to random error resulting from sampling variation, typically represented by the width of the confidence interval. This type of error affects the reliability of individual results rather than systematically skewing them.
2. Quality of a study is not always directly correlated with bias. Quality is difficult to define and very much context-dependent; for practical purposes a methodological quality may be applied, e.g., as adherence to guidelines and best practices. Even very high-quality studies can be biased because in some research contexts bias can be effectively impossible to eliminate and not all flaws necessarily introduce bias.
3. Reporting emphasizes that good methodologies can still fail to be adequately communicated. Inadequate reporting may obscure the true quality of a study, complicating the assessment of potential biases and replicability.

**Fig. 1:**
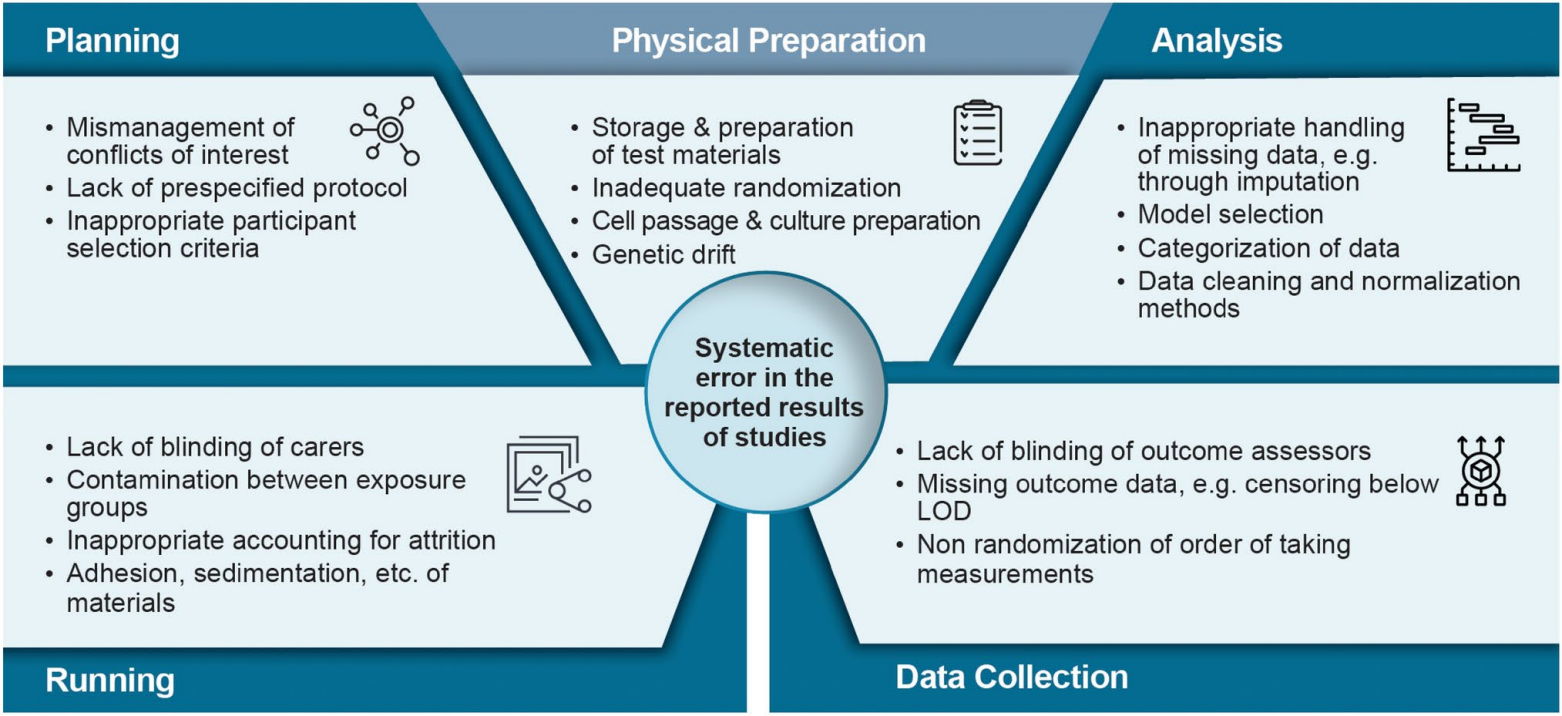
Sources of systematic error in toxicological studies. Non-comprehensive illustration of possible introductions of systematic errors possibly leading to bias

In the context of toxicological studies, it is essential to differentiate these concepts to ensure appropriate risk assessments, where bias assessments focus on systematic errors, distinct from random errors or study design and reporting deficiencies. In the field of toxicology, where studies often inform regulatory decisions and public health policies, it is particularly important to identify and account for potential biases that may influence the reliability and validity of study findings. Toxicological studies, whether in vivo animal studies, in vitro mechanistic studies, or human observational studies, are subject to various sources of bias that can compromise their internal validity. By systematically assessing the risk of bias in individual studies (Fig. 1), researchers can determine the confidence in the study results and the weight they should be given in the overall body of evidence.

Moreover, assessing the risk of bias can help understand the discrepancies in results across studies. Studies with higher risk of bias may yield different results than those with lower risk, contributing to heterogeneity in the evidence base. By identifying the sources of bias and their potential impact, researchers can better interpret the variability in study findings and make more informed judgments about the consistency and coherence of the evidence.

Furthermore, evaluating the risk of bias in published studies can inform the design and conduct of future research. By identifying the common pitfalls and limitations of previous studies, researchers can take steps to minimize bias in their own work, such as using appropriate randomization and blinding procedures, prespecifying outcomes and analysis plans, and ensuring complete and transparent reporting. Thus, the widespread adoption of risk of bias assessment can ultimately improve the quality and credibility of the toxicological literature.

Here, we will delve into the concept of risk of bias and its importance in evidence-based toxicology (Hartung and Tsaioun 2024). We will discuss the main types of bias that can affect toxicological studies, the tools and frameworks available for assessing risk of bias, and the implications of bias assessment for evidence synthesis and decision-making. By understanding and addressing the risk of bias, toxicologists can strengthen the foundation of evidence-based toxicology and enhance the reliability and impact of their work.

Risk of bias is the evaluation of the potential for systematic errors in the outcomes or interpretations of a study, leading to conclusions that deviate from the true effect of an intervention or exposure. While initially developed in the context of clinical trials, risk of bias assessments have become essential in other research fields, including toxicology, where diverse study designs are employed to assess chemical safety and biological effects (Higgins et al. 2011; Frampton et al. 2022). The importance of risk of bias evaluation is underscored by the increasing use of toxicological data in regulatory decision-making, such as chemical risk assessments for public health and environmental safety (Klimisch et al. 1997; Vesterinen et al. 2014; Whaley et al. 2016; Wikoff et al. 2018).

Historically, quality assessment of toxicological studies has relied on checklists or scoring systems that consider reporting quality and adherence to guidelines (Samuel et al. 2016). However, these approaches often fail to capture the nuanced biases that can arise at different stages of study design and implementation (Hooijmans et al. 2014), while conflating issues of reporting quality and imprecision with systematic error. The risk of bias tools aim to provide a more structured and systematic approach by focusing exclusively on internal validity issues, breaking them down into a set of domains of bias that comprehensively cover factors that may introduce systematic errors into a study. This shift from quality scoring to bias assessment represents a paradigm change, aligning toxicology with the principles of evidence-based medicine (Ritskes-Hoitinga and Wever 2018).

The risk of bias concepts are highly relevant not only to toxicological research but also to broader areas of basic research and product development such as drug discovery. Bias in these contexts can have profound implications for the reliability of experimental results, the validity of mechanistic conclusions, and the safety and efficacy profiles of potential therapeutic agents. Understanding and mitigating risk of bias in basic research and product development is essential for ensuring that scientific findings are robust, reproducible, and translatable to clinical applications. While this article explores the application of risk of bias concepts in toxicology, most considerations seem relevant also across these domains.

## Bias (types) and key distinctions

### Understanding bias and risk of bias

Bias is a systematic distortion in research findings, i.e., estimations of effect, association, or inference.^1^ These distortions mean there are differences between the reported findings of a study and the truth. The bias results from limitations in the design, conduct, analysis, or reporting of a study and exists whether or not we can detect it, i.e., bias is theoretically measurable but not always detectable. A major reason is the lack to adherence to reporting standards (Kilkenny et al. 2010; Hartung et al. 2019). Bias is often not detectable, or at least measurable, because usually too few studies conducted with sufficiently similar designs and limitations are available to determine using observational methods how much bias a given limitation might introduce (MacLeod et al. 2004; Metelli and Chaimani 2020). While controlled trials of how much bias a given limitation might introduce are challenging but theoretically possible to conduct (Haber et al. 2024), this is not the same thing as measuring how biased any given study might be.

While true bias resists empirical measurement, risk of bias can, in contrast, be anticipated. Risk of bias represents an assessment of the likelihood that bias exists in a study, based on observable features of study design and reporting. Ideally, there is empirical evidence that these features can introduce bias, but there should at least be good theoretical reason for thinking a feature would introduce bias. The concept of risk of bias is, therefore, fundamentally different from bias itself, as it is a predictive assessment of potential for bias based on methodological indicators, rather than a direct measurement of bias itself. The assessment is inherently probabilistic and limited by the information available in study reports and protocols.

This distinction carries important implications for AI approaches in two key areas. First, when modeling bias itself, AI requires access to ground truth data and can potentially measure actual systematic errors. These approaches may identify novel indicators of bias and are particularly suited for machine learning approaches that can detect patterns in outcomes. Second, when assessing risk of bias, AI systems must follow predefined assessment criteria based on methodology reporting and rely on proxy indicators. This approach is well-suited for rule-based automation but remains constrained by traditional assessment frameworks.

Understanding this distinction helps frame two different potential roles for AI: automating traditional risk of bias assessments and developing new approaches to detecting actual bias through pattern recognition. Each role requires different methodological approaches and has distinct limitations and opportunities.

### Types of bias

**Fig. 2:**
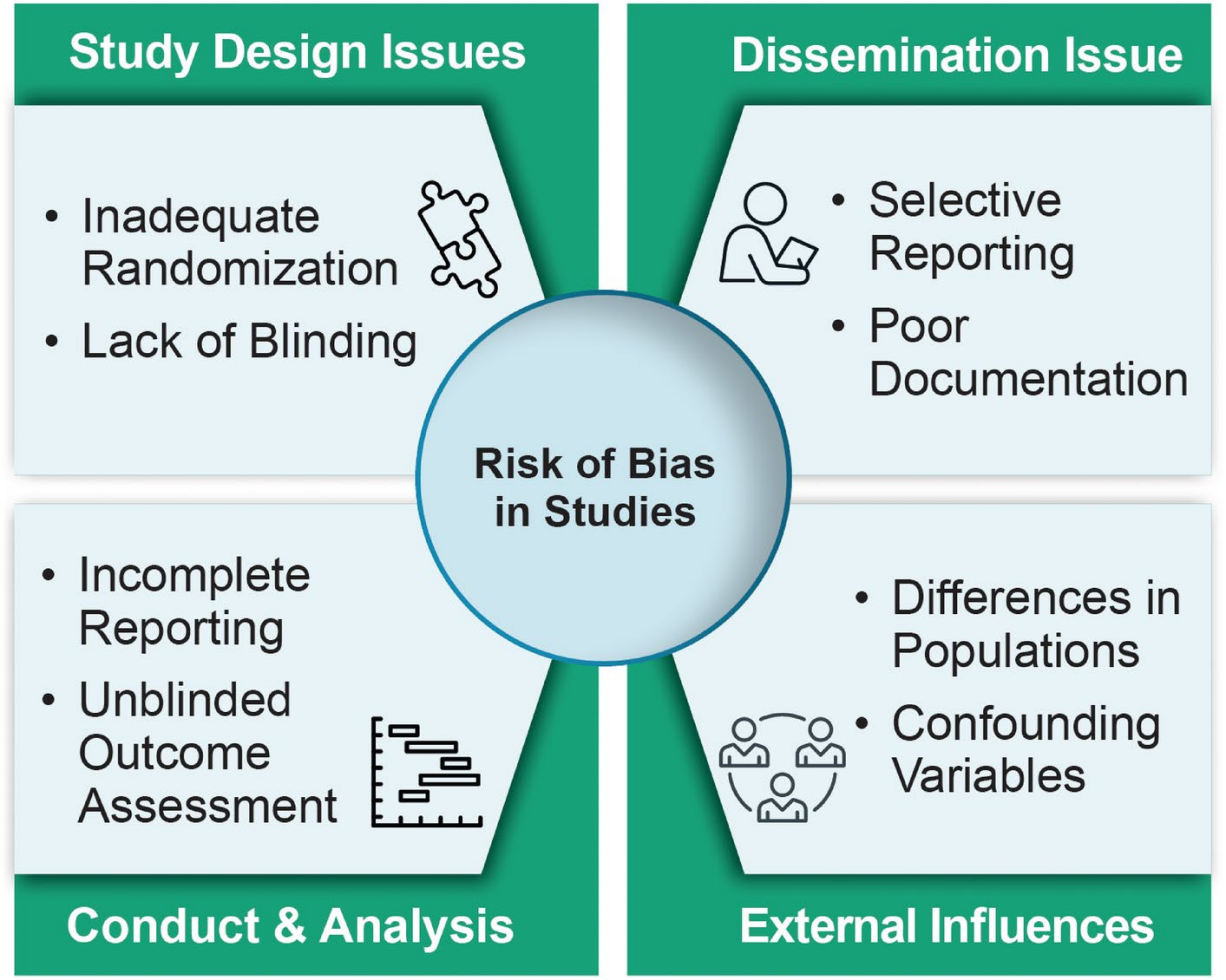
The main types of bias relevant to toxicological studies

There are several key types of bias that can affect toxicological studies (Fig. 2), each relating to different aspects of the study design, conduct, analysis, or reporting. While the terminology and categorization of biases may vary across different frameworks and tools, five of the main types of bias relevant to toxicology include:

1. *Selection bias*: selection bias arises from systematic differences between baseline characteristics of the groups being compared, leading to groups that are not truly comparable. In toxicological studies, selection bias can occur due to inadequate randomization of animals or cell cultures to treatment groups, or due to differences in the source or handling of the test systems. For example, if animals are allocated to treatment groups based on their weight or activity level, rather than randomly, this can introduce systematic differences between the groups that may confound the treatment effect. Similarly, if cell cultures are not properly randomized to plates or wells, differences in cell density or passage number may bias the results. Selection bias can also occur in observational studies, where the exposure groups may differ in their characteristics or risk factors, leading to spurious associations or masking of true effects. For instance, if a study compares workers exposed to a chemical to a general population sample, differences in age, sex, or socioeconomic status between the groups may bias the observed association between the exposure and health outcomes. To minimize selection bias, it is crucial to use proper randomization and allocation procedures, such as computer-generated random sequences with group allocation decisions not under control of the investigators. Adequate reporting of the randomization and allocation process is also important as it is used for assessing the risk of selection bias.
2. *Performance bias*: performance bias refers to systematic differences in the intended exposure or intervention, and the actual exposure or intervention received by the groups in a study. These differences can include care or exposure of groups to factors other than the intervention of interest. Performance bias can arise from differences in the housing, feeding, or handling of animals, in the preparation or dosing of treatments, in adherence to an intervention, or differences in participant behavior between exposure or intervention groups. For example, if animals in a treatment group receive more frequent handling or attention from the investigators, this may lead to differences in stress levels or behaviors that can affect the outcome measures. Human participants known to be in a control group may receive a different standard of care during a study, and interventions that have side-effects or may otherwise be more difficult to adhere to may result in different levels of adherence between groups. In observational studies, performance bias can occur if the exposure groups differ in their access to health care, lifestyle factors, or other exposures that may influence the outcomes, though these might also be seen just as cofounders. For instance, if workers exposed to a chemical also have higher rates of smoking or alcohol consumption, this may confound the association between the chemical exposure and health effects. Blinding of participants, animals or samples and personnel to the treatment allocation is a key strategy for minimizing performance bias. In animal studies, this may involve using coded labels for treatment and control substances, and ensuring that animal caretakers and investigators are unaware of the group allocation. This applies similarly to the wells of an in vitro study. Blinding may not always be practical or possible, for example, caretakers in animal studies will be able to correlate severity of clinical effects with administered doses or caretakers cannot be blind due to animal welfare requirements (Karp et al. 2022), but performance bias always matters nonetheless.
3. *Detection bias*: detection bias arises from systematic differences in how outcomes are assessed between groups. In toxicological studies, detection bias can occur if the investigators measuring the outcomes are aware of the treatment allocation, and this knowledge influences their measurements or judgments. For example, if a pathologist evaluating tissue slides knows which animals were in the treatment group, they may be more likely to detect or score lesions in those animals, leading to an overestimation of the treatment effect. Detection bias can also occur if the methods used to assess outcomes differ between groups, or if there are differences in the timing or frequency of assessments. For instance, if animals in the treatment group undergo more frequent or intensive monitoring than control animals, this may lead to the detection of more subtle or transient effects in the treated group. To minimize detection bias, it is important to blind the investigators assessing the outcomes to the treatment allocation, using coded labels or samples whenever possible. Standardizing the outcome assessment methods and timing across all groups, and using objective and validated measures where available, can also help reduce detection bias. Adequate reporting of the blinding procedures and outcome assessment methods is crucial for evaluating the risk of detection bias.
4. *Attrition bias*: attrition bias, also known as exclusion bias or loss-to-follow-up bias, arises from systematic differences between groups in withdrawals or exclusions from the study. In toxicological studies, attrition bias can occur if animals or samples are lost, excluded, or censored from the analysis in a non-random manner, leading to incomplete outcome data. For example, if animals in the treatment group are more likely to die or be euthanized due to toxicity, and these animals are excluded from the analysis, this can lead to an underestimation of the treatment effect.

Attrition bias can also occur in observational studies if there are differences in the rates or reasons for loss to follow-up between the exposure groups. For instance, if participants with higher exposures or more severe health outcomes are more likely to drop out of the study, this can lead to a distortion of the exposure–response relationship.

To minimize attrition bias, it is important to strive for complete outcome data for all subjects, and to analyze all subjects according to their original treatment allocation, regardless of adherence or withdrawal (i.e., intention-to-treat analysis). If exclusions or censoring are unavoidable, the reasons should be clearly reported and balanced across groups. Sensitivity analyses can be used to assess the impact of missing data on the results, using different assumptions or imputation methods.

5. *Reporting bias*: reporting bias refers to the selective reporting of outcomes or analyses based on the nature or direction of the results. Reporting bias is very important, but not because it biases individual findings necessarily, but because it distorts the evidence base through selective omission of outcomes (reporting bias, on its own, does not make the outcomes that are reported any less true). Sterne (2013) argues quite convincingly that reporting bias should be treated as dissemination bias. Noteworthy, GRADE^2^ is also updating its guidance, separating risk of bias as internal validity from dissemination bias, of which reporting bias is one type (a study may selectively report its results, but it does not mean those results are not true). In toxicological studies, reporting bias can occur if investigators selectively report statistically significant or “positive” findings, while omitting non-significant or “negative” results. This can lead to an overestimation of the magnitude or consistency of the treatment effects in the literature. Reporting bias can also occur if investigators selectively report subgroup analyses, secondary outcomes, or post-hoc analyses that were not prespecified in the study protocol. This can lead to “data dredging” or “p-hacking”, where multiple analyses are conducted until a significant result is found, leading to spurious associations. To minimize reporting bias, it is crucial for investigators to prespecify the primary and secondary outcomes, as well as the analysis plan, in a study protocol or registration. Any deviations from the prespecified plan should be justified and reported transparently. Reporting all outcomes and analyses, regardless of statistical significance, and using standardized reporting guidelines (e.g., ARRIVE for animal studies) can also help reduce reporting bias.

**Fig. 3:**
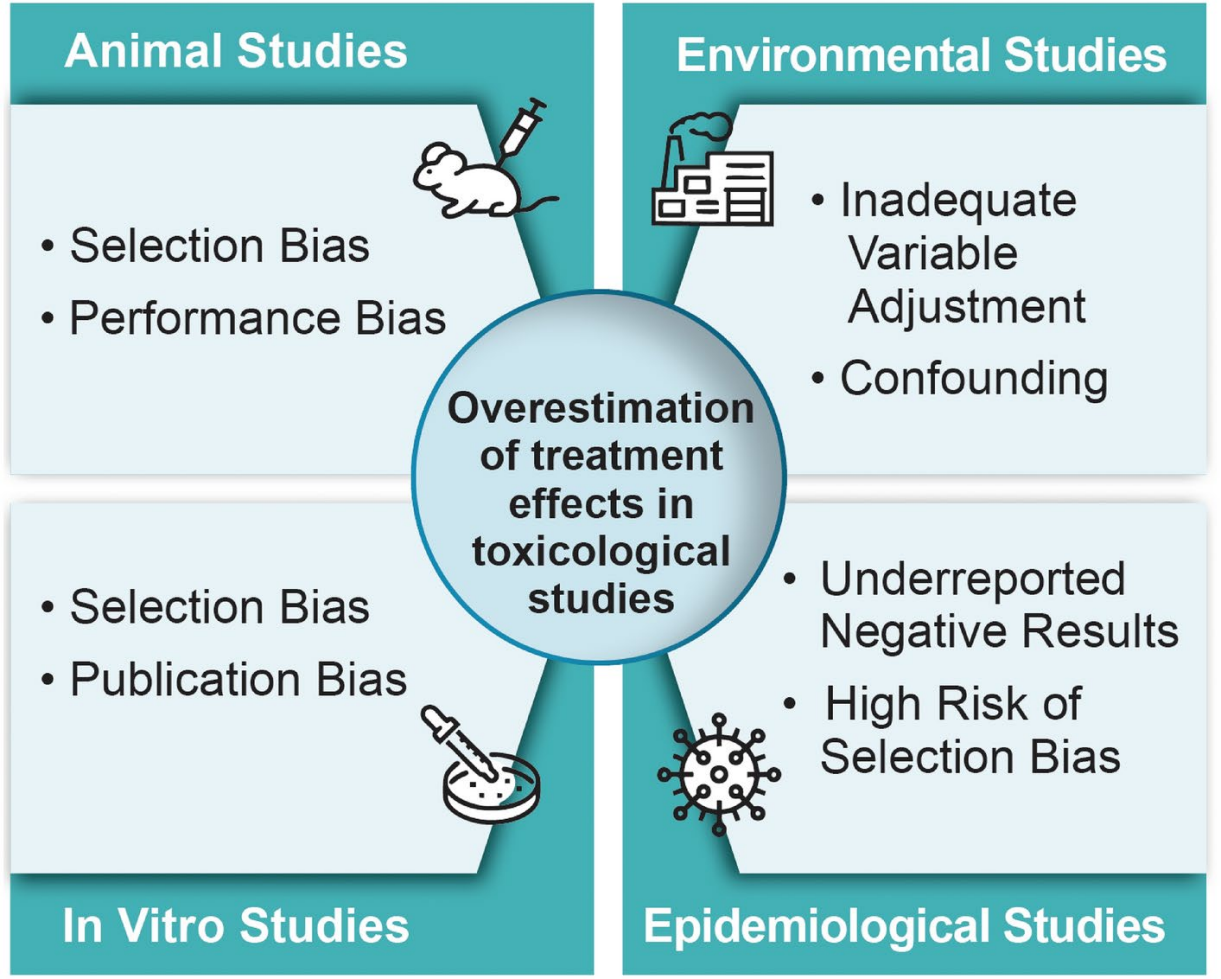
Overview of common types of bias in different types of toxicological research. A conceptual diagram that visually categorizes the main types of bias encountered in toxicological studies, such as selection bias, performance bias, detection bias, attrition bias, and reporting bias. This provides a foundational understanding of the various biases and their relevance to toxicological research

By understanding and assessing these different types of bias (Fig. 3), toxicologists can better evaluate the internal validity of individual studies and the overall quality of the evidence base. Noteworthy, there are more categories of bias, for example, for the INVITES-IN tool for assessing the internal validity of in vitro studies, we used ten (Mathisen et al. 2024). This, in turn, can inform the design and conduct of future studies, as well as the interpretation and application of the evidence for decision-making.

### Direction and magnitude of bias: empirical evidence

The direction of bias refers to whether systematic errors tend to overestimate or underestimate the true effect of an intervention or exposure. Understanding bias direction is crucial for interpreting study results and their implications for regulatory decisions. While risk of bias tools typically assess the likelihood of bias presence, they often do not predict its direction or magnitude. In the Cochrane Handbook,^3^ predicting direction is discouraged without evidence for direction. There is, in some circumstances, evidence that, e.g., not blinding inflates effect size, but this is probably not the general case. A limitation in a study will result in bias that goes in the direction of the expected or desired result. So, if a team wants an intervention to work, they will observe an increase in effect size. The empirical evidence from meta-research has revealed consistent patterns in how different types of bias influence study outcomes.

- *Selection bias*: meta-analyses of toxicological studies have demonstrated that inadequate randomization typically leads to overestimation of treatment effects. For example, Krauth et al. (2013) found that studies with high risk of selection bias reported effect sizes approximately 30% larger than properly randomized studies. This inflation occurs because researchers may unconsciously assign animals with better baseline characteristics to treatment groups, creating an artificial advantage that manifests as increased efficacy.
- *Performance bias*: the direction of performance bias often depends on researcher expectations. Studies by Macleod et al. (2015) showed that lack of blinding in animal studies led to a 27% overestimation of treatment effects. This bias tends to align with researcher hypotheses—positive effects are inflated in studies of beneficial interventions, while adverse effects are potentially underestimated in toxicological assessments. A systematic review by Hooijmans et al. (2014) found that unblinded toxicology studies were more likely to miss subtle adverse effects, particularly in behavioral assessments.
- *Detection Bias*: Empirical investigations have revealed that unblinded outcome assessment consistently inflates effect sizes. Sena et al. (2010) demonstrated that studies with unblinded outcome assessment reported efficacy measures 34% larger than blinded studies. In toxicology specifically, a meta-analysis by Vesterinen et al. (2014) found that unblinded studies underestimated adverse effects by 15–25%, particularly for subjective outcomes like histopathological assessments.
- *Publication bias*: the direction of publication bias typically favors positive or statistically significant results. A comprehensive analysis by Bero et al. (2016) of toxicological studies showed that:

- Positive findings were 3.3 times more likely to be published than negative results
- Studies showing adverse effects had longer time to publication
- Industry-funded studies showing adverse effects were less likely to be published than independently funded studies with similar findings

A key question is the magnitude of combined biases. When multiple sources of bias are present, their effects can be additive or multiplicative. A meta-analysis by Hirst et al. (2014) found that studies with multiple high risk of bias indicators overestimated effect sizes by 40–60% compared to studies with low risk of bias across all domains. In toxicology specifically, studies with high risk of bias in multiple domains underestimated adverse effects by up to 48% (Rooney et al. 2016).

### Implications for AI-based assessment

Understanding these empirical patterns of bias direction has important implications for AI-based approaches:

- *Training data considerations*: meta-research findings can inform the development of AI models by providing empirical priors about the expected direction and magnitude of bias under different methodological conditions. This knowledge can help calibrate machine learning models and improve their predictive accuracy.
- *Novel pattern detection*: AI systems might identify previously unknown combinations of study characteristics that predict specific directions of bias. For example, machine learning analyses of large datasets might reveal interaction effects between different sources of bias that are not apparent in traditional meta-analyses.
- *Validation metrics*: when developing AI tools for bias assessment, performance metrics should consider not only the detection of bias presence but also the accuracy in predicting bias direction and magnitude. This requires careful consideration of ground truth data and validation approaches, but there might not be enough studies to provide this grounding. We have recently suggested to model validation studies with AI tools (Hartung et al. 2024), which might help here.
- Context-specific adjustments: the empirical evidence suggests that bias patterns may vary across different fields of toxicology and types of outcomes. AI systems should be capable of adjusting their assessments based on these context-specific patterns, rather than applying one-size-fits-all corrections.

This empirical understanding of bias direction and magnitude is essential for developing more sophisticated AI approaches to bias assessment. Rather than simply flagging potential sources of bias, next-generation AI tools could provide quantitative estimates of likely bias direction and magnitude, i.e., to treat risk of bias as probabilistic and prognostic/predictive, enabling more nuanced interpretation of study results and better-informed regulatory decisions.

## Tools for assessing risk of bias

To facilitate systematic and transparent assessment of bias, several tools have been developed. Each of these tools provides a structured approach to evaluate bias within distinct domains of study design, conduct, analysis, and reporting (Higgins et al. 2011). While initially developed for clinical research, tools like the Cochrane Risk of Bias Tool have been adapted for use in toxicology, for example, by SYRCLE’s Risk of Bias Tool for animal studies (Hooijmans et al. 2014), and the Office of Health Assessment and Translation (OHAT) Risk of Bias Tool (OHAT 2019).

1. *Cochrane risk of bias tool*: the Cochrane Risk of Bias Tool, developed by the Cochrane Collaboration, is one of the most widely used tools for assessing risk of bias in randomized controlled trials (RCTs). The tool covers seven domains of bias: random sequence generation, allocation concealment, blinding of participants and personnel, blinding of outcome assessment, incomplete outcome data, selective reporting, and other sources of bias. For each domain, the tool provides a series of signaling questions to guide the assessment, and a judgment of low, high, or unclear risk of bias (Higgins et al. 2011). The assessment is based on the information reported in the study, and the reviewer's judgment of the likelihood of bias. The tool also includes a space for free text comments to justify the judgment, e.g., by providing quotes from the study. The Cochrane Risk of Bias Tool has been widely validated and has been shown to have good inter-rater reliability and construct validity. Although primarily designed for randomized controlled trials, it has been adapted for non-randomized studies, including observational research (Sterne et al. 2016). Noteworthy, that the Cochrane Risk of Bias tool has been updated^4^ (including reduction to five bias domains and fixed response options to signaling questions) (Higgins et al. 2019) but updates of the offsprings for toxicology may take some time.
2. *SYRCLE's Risk of Bias Tool*: The SYstematic Review Center for Laboratory animal Experimentation (SYRCLE) has developed a risk of bias tool specifically for animal intervention studies, based on the Cochrane Risk of Bias Tool. The SYRCLE's Risk of Bias Tool (Hooijmans et al. 2014) covers the same seven domains of bias as the Cochrane tool but provides signaling questions and examples that are specific to animal studies. It includes animal-specific considerations such as adequacy of the animal model, the timing of randomization and choice of control group. For example, in the domain of blinding of personnel, the tool asks whether the caregivers and investigators were blinded to the intervention, and provides examples of low risk (e.g., identical looking treatments, coded cages), high risk (e.g., different looking treatments, open-label study), and unclear risk (e.g., no information on blinding). The tool has been validated in a sample of animal studies and has been shown to have good inter-rater agreement and construct validity (Hooijmans et al. 2014). A recent study by van Luijk et al. (2021) demonstrated that applying SYRCLE’s tool to a cohort of animal studies improved the consistency and transparency of risk of bias assessments compared to unstructured evaluations.

3. *OHAT Risk of Bias Tool*^5^ (OHAT 2019): the Office of Health Assessment and Translation (OHAT) at the National Institute of Environmental Health Sciences (NIEHS) of the US NIH has developed a risk of bias tool for environmental health studies, which covers a range of study designs, including human observational studies, animal studies, and in vitro mechanistic studies. The OHAT tool assesses risk of bias in seven domains: selection, confounding, performance, attrition/exclusion, detection, selective reporting, and other sources of bias. For each domain, the tool provides a series of questions to guide the assessment, and a rating of low, probably low, probably high, or high risk of bias. The ratings are based on the severity and direction of the potential bias, and the reviewer's judgment of the likelihood of bias. It uses a common set of criteria for seven domains of bias and applies domain-specific criteria depending on the study design. This flexibility makes the OHAT tool well-suited for environmental health research, where diverse study types are often synthesized (Rooney et al. 2014). The tool has been employed in systematic reviews of environmental contaminants, such as perfluoroalkyl substances (PFAS) and their effects on immune and developmental health (Koustas et al. 2014) and the effects of traffic-related air pollution on respiratory health. One notable feature of the OHAT tool is that it assesses risk of bias on an outcome-specific basis, recognizing that the potential for bias may differ across outcomes within a study. For example, a study may have a low risk of detection bias for objective outcomes (e.g., mortality), but a high risk of detection bias for subjective outcomes (e.g., behavior). The OHAT tool also emphasizes the importance of considering the direction and magnitude of potential bias, not just the presence or absence of bias indicators. For example, a study with a small amount of attrition may still have a low risk of attrition bias if the reasons for attrition are unlikely to be related to the outcome, and the attrition is balanced across groups. The tool has been adapted for use in systematic reviews of mechanistic data, such as high-throughput screening assays and toxicogenomic studies.^6^

### Related quality assessment tools in toxicology

While tools like the Cochrane and OHAT Risk of Bias frameworks (Table 1) have been widely adopted, the unique challenges of toxicological research necessitate the use of specialized tools such as the ToxRTool,^7^ SciRAP,^8^ and the Navigation Guide.^9^ These tools have been developed or adapted to address the methodological intricacies of toxicology studies, including variable exposure conditions, complex outcomes, and diverse study designs. The ToxRTool, for instance, provides detailed criteria for evaluating the reliability of both in vitro and in vivo toxicological data (Schneider et al. 2009), while the SciRAP tool incorporates relevance and applicability assessments for regulatory risk assessment (Larsson et al. 2018). The Navigation Guide, in contrast, is particularly well-suited for synthesizing environmental health research, as it integrates risk of bias assessments with frameworks for evidence integration and policy application (Woodruff and Sutton 2014).

4. *ToxRTool (Toxicological data Reliability Assessment Tool)*: developed by the European Centre for the Validation of Alternative Methods (ECVAM), the ToxRTool was designed to evaluate the reliability and quality of in vitro and in vivo toxicological studies. The tool provides criteria to assess both methodological and reporting quality, focusing on aspects such as adherence to standard protocols, sample size, and data completeness. ToxRTool categorizes studies into three reliability categories, aka the “Klimisch classes”: “reliable without restrictions,” “reliable with restrictions,” and “not reliable,” based on a scoring system (Schneider et al. 2009).
5. *Navigation guide*: developed as a systematic review methodology for environmental health research (Woodruff and Sutton 2014), the Navigation Guide integrates principles from systematic reviews used in clinical research and adapts them for environmental and toxicological studies. The methodology involves four steps: specifying the study question, selecting the studies, assessing the risk of bias, and synthesizing evidence to reach a conclusion on the strength of evidence (Woodruff and Sutton 2014). The Navigation Guide has been applied to evaluate the health effects of various environmental exposures, such as

**Table 1:**
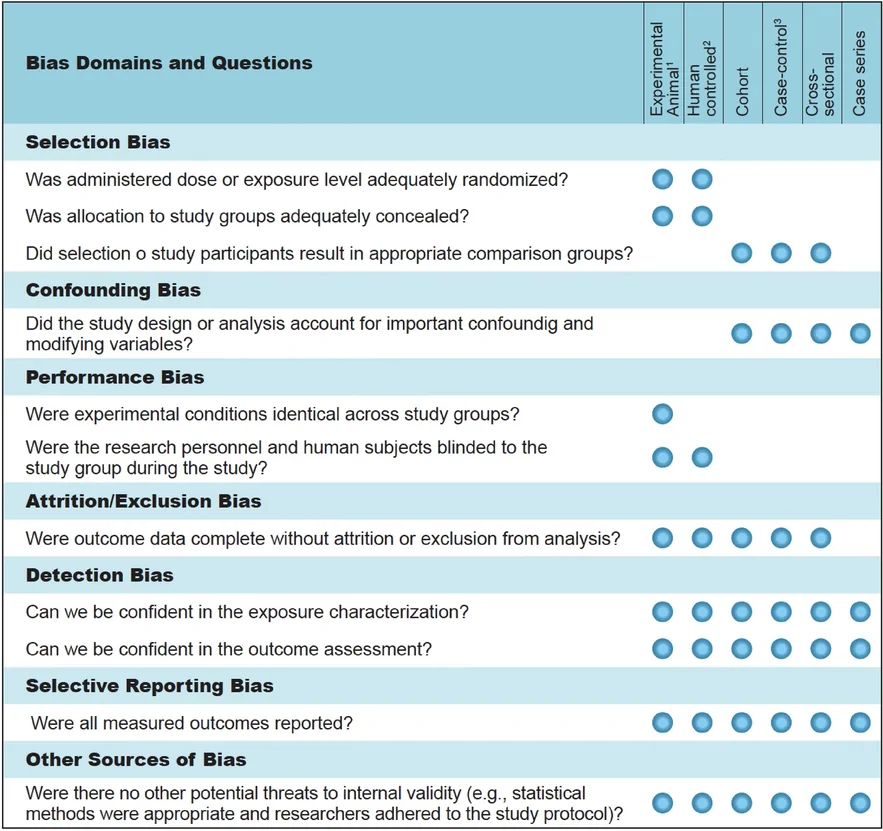
OHAT Parallel Risk of Bias approach

Modified from the presentation “*Extending a Risk-of-Bias Approach to Address In Vitro Studies*” by Dr. Andrew Rooney, National Toxicology Program, Office of Health Assessment and Translation, Dec 17, 2015, available at https://ordspub.epa.gov/ords/eims/eimscomm.getfile?p_download_id=526750

^1^Experimental animal studies are controlled exposure studies. Non-human animal observational studies could be evaluating using the design features of observational human studies such as across-sectional study design

^2^Human controlled trials (HCTs): studies in humans with a controlled exposure, including randomized controlled trials (RCTs) and non-randomized experimental studies

^3^Cross-sectional studies include population surveys with individual data (e.g., NHANES) and population surveys with aggregate data (i.e., ecological studies)

The Navigation Guide has been applied in several case studies to evaluate the health effects of environmental exposures: perfluorooctanoic acid (PFOA) and fetal growth (Johnson et al. 2014), developmental exposure to ambient particulate air pollution and birth weight (Uwak et al. 2021), air pollution and autism spectrum disorder (Lam et al. 2016), polybrominated diphenyl ethers (PBDEs) and neurodevelopment (Lam et al. 2017) as well as formaldehyde exposure and asthma outcomes (Lam et al. 2021).

6. *SciRAP (Science in Risk Assessment and Policy)*: SciRAP is a web-based tool developed to assess the reliability and relevance of in vitro and in vivo toxicological studies for chemical risk assessment. The tool includes two evaluation templates: one for in vivo studies and another for in vitro studies, each with criteria related to study design, reporting, and data integrity (Larsson et al. 2018). SciRAP has been used to support chemical risk assessments in regulatory contexts, making it a practical tool for environmental health research.

While these tools differ in their scope, structure, and terminology, they share a common goal of providing a systematic and transparent approach for evaluating the internal validity of studies. When selecting a risk of bias tool for a particular systematic review or evidence assessment, it is important to consider the specific research question, the types of studies included, and the feasibility and resources available for the assessment (Frampton et al. 2022). It may also be necessary to adapt or modify existing tools to fit the specific context or requirements of the review. Regardless of the specific tool used, the key principles of risk of bias assessment remain the same: to identify the potential sources of bias in each study, to evaluate the likelihood and magnitude of bias based on the information reported, and to use the risk of bias judgments to inform the overall quality and certainty of the evidence. By applying these principles consistently and transparently, toxicologists can strengthen the foundation of evidence-based toxicology and improve the reliability and impact of their work.

The various tools for assessing risk of bias share several common core assessment domains. The first domain encompasses fundamental study design elements: the implementation of randomization procedures, the extent of blinding, the selection and composition of control groups, and the determination of appropriate sample sizes. These elements form the foundation of study validity and reliability.

The second domain focuses on implementation features, including adherence to established protocols, the robustness of data collection methods, the presence and effectiveness of quality control measures, and the systematic handling of protocol deviations. These features help ensure the study’s execution aligns with its design and maintains scientific rigor throughout the research process.

The third domain addresses analysis and reporting, encompassing the appropriateness of statistical methods, the completeness of outcome reporting, the handling of missing data, and the transparency of documentation. This domain is crucial for evaluating the reliability of study conclusions and the reproducibility of research findings.

This commonality across tools suggests significant opportunities for standardized AI approaches while highlighting essential methodological features that should be machine-accessible for automated assessment.

## Importance of assessing risk of bias

Assessing the risk of bias in individual studies is a crucial step in the systematic review and evidence assessment process, with important implications for the interpretation, synthesis, and application of the evidence. Ioannidis (2005) discusses how various biases, including reporting bias, can lead to false-positive findings in research and is highly relevant to the discussion on the importance of assessing risk of bias in toxicological studies. Macleod et al. (2015) provides empirical evidence on the prevalence of various biases in animal research, supporting the discussion on how bias can affect study outcomes and the overall reliability of the evidence base.

There are several key reasons why assessing risk of bias is essential for evidence-based toxicology:

1. *Determining the reliability and validity of study findings*: the primary goal of risk of bias assessment is to evaluate the internal validity of each study, or the extent to which the study design, conduct, analysis, and reporting minimize the potential for bias. The studies with a high risk of bias in one or more domains may have compromised internal validity, meaning that the observed results may not accurately reflect the true effect of the intervention or exposure. By systematically assessing the risk of bias, researchers can determine the level of confidence they can place in the findings of each study, and the weight they should be given in the overall body of evidence. For example, a study with a high risk of selection bias due to inadequate randomization or allocation concealment may have imbalanced baseline characteristics between groups, leading to confounding and distortion of the observed effect. Similarly, a study with a high risk of detection bias due to unblinded outcome assessment may have inflated or biased effect estimates, particularly for subjective outcomes. By identifying these potential sources of bias, researchers can adjust their interpretation of the study results and consider them in the context of other, less biased studies.
2. *Explaining heterogeneity in results across studies*: another important reason for assessing risk of bias is to understand and explain the variability or inconsistency in results across studies. Studies with different risks of bias may yield different effect estimates, even if they are investigating the same research question. By examining the risk of bias profiles of studies with discordant results, the researchers can identify potential sources of heterogeneity and explore whether the differences in results are likely due to bias or other factors, such as differences in populations, interventions, or outcomes. For example, if a meta-analysis of animal studies on the effects of a chemical on liver toxicity finds significant heterogeneity in effect sizes, the researchers may examine whether studies with a high risk of performance bias (e.g., due to lack of blinding of personnel) tend to have larger effect sizes than studies with a low risk of performance bias. If this is the case, the researchers may conclude that the observed heterogeneity is likely due to bias, rather than true differences in the chemical's effects across studies.
3. *Informing the design of future studies*: assessing the risk of bias in published studies can also provide valuable lessons for the design and conduct of future research. By identifying the most common sources of bias and the study design features that are associated with lower risks of bias, the researchers can improve the quality and reliability of their own studies. This can involve using appropriate randomization and allocation concealment methods, blinding personnel and outcome assessors, prespecifying outcomes and analysis plans, and ensuring complete and transparent reporting. For example, if a systematic review of in vitro studies on the genotoxicity of a chemical finds that studies using coded samples and blinded outcome assessment have lower risks of detection bias and more consistent results than studies using open-label designs, this may prompt researchers to adopt these best practices in their own in vitro genotoxicity studies.

Each study type requires a tailored approach to risk of bias assessment to account for these unique sources of bias. Understanding these differences enables more accurate evaluation of study validity and reliability when conducting systematic reviews or risk assessments in toxicology. The differences in risk of bias for in vivo, in vitro, and in silico studies (Table 2) arise from the distinct methodologies and experimental designs associated with each type of research. Understanding these differences is crucial for properly assessing the quality and validity of studies in toxicology. Here’s an overview of the main distinctions between the risk of bias in these study types based on author’s experiences, though there is not much hard evidence for relative importance of bias against study design for any domain:

**Table 2:**
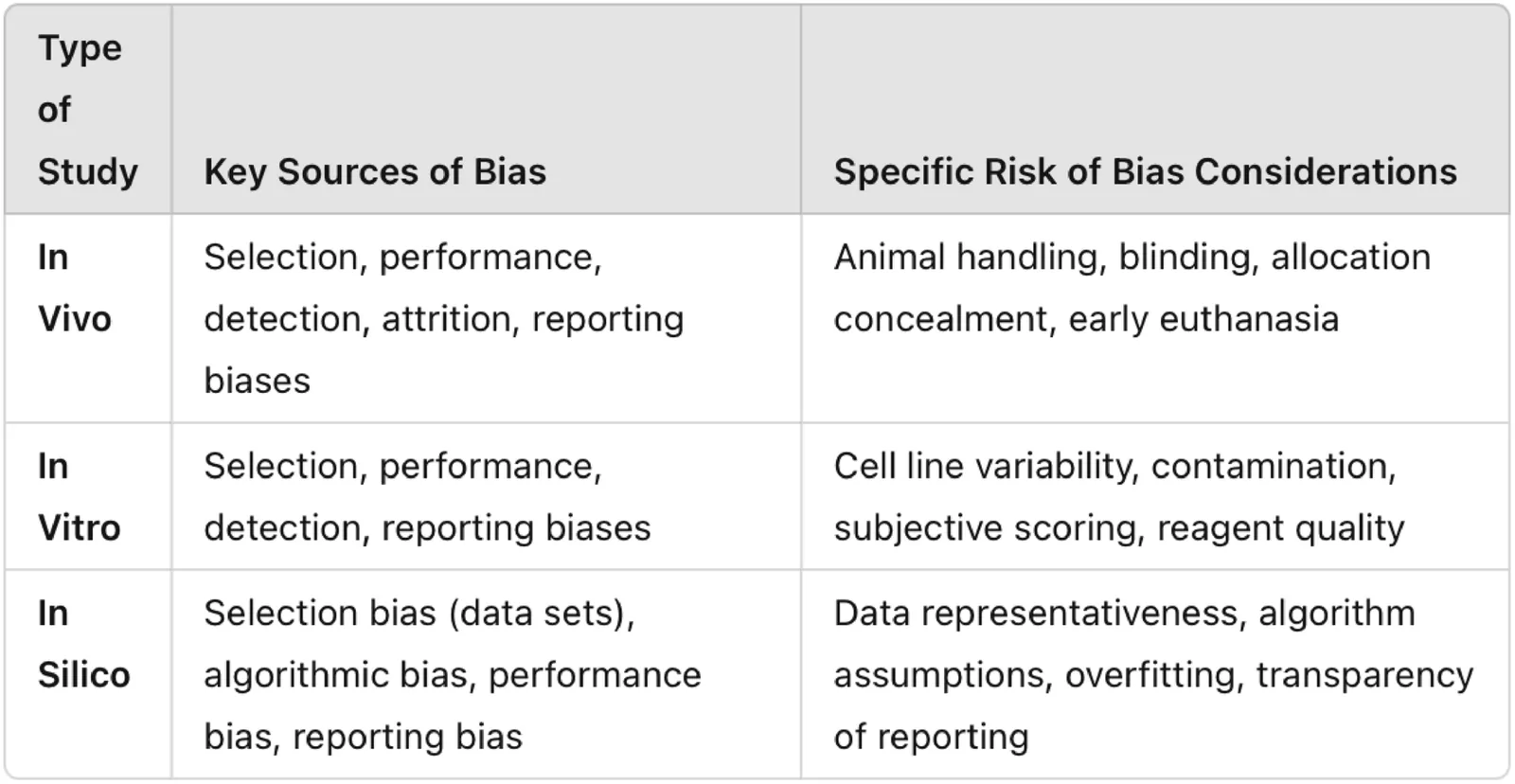
Differences in risk of bias for in vivo, in vitro, and in silico studies

### In vivo studies

In vivo studies involve research conducted within living organisms, typically animals, and can include studies on exposure to chemicals, drugs, or other environmental agents. Due to their complexity, in vivo studies are susceptible to several types of bias that are generally not as relevant for in vitro or in silico studies.

Key risk of bias types:

- *Selection bias*: occurs if animals are not randomly assigned to treatment and control groups. This bias can result from non-random allocation of animals based on weight, sex, or health status.
- *Performance bias*: arises from systematic differences in care or handling between groups, such as differences in housing conditions, feeding, or interaction with researchers.
- *Detection bias*: results from knowledge of group allocation affecting outcome assessment. For instance, if researchers know which animals are in the treatment group, they may consciously or unconsciously record observations differently.
- *Attrition bias*: occurs if animals drop out or are excluded non-randomly, which can alter the balance between groups. This bias often arises due to adverse effects in the treatment group leading to early euthanasia.
- *Reporting bias*: results from selective outcome reporting based on the nature of the findings (e.g., publishing only positive results) (Sena et al. 2010).

Special considerations: in vivo studies are particularly susceptible to biases due to variations in environmental factors, animal handling, and inter-animal variability, which can significantly influence results. Blinding, randomization, and adequate sample size are critical in reducing these biases. Noteworthy, the most extensive quality assurance scheme, OECD’s Good Laboratory Practice,^10^ has been developed around animal testing, however, it is less suited to non-regulatory settings, particularly academia; for example, it requires the execution of work by fully trained personnel, a dedicated workplace for each test and enormous documentation/auditing efforts. Furthermore, guidance is available from organizations such as the American Association for Laboratory Animal Science (AALAS)^11^ and Federation of European Laboratory Animal Science Associations (FELASA).^12^

### In vitro studies

In vitro studies involve research conducted outside of a living organism, typically in cell cultures, tissue samples, or biochemical assays. Although these studies are more controlled than in vivo research, they are still prone to specific types of bias.

Key risk of bias types

- *Selection bias*: occurs if cells or tissue samples are not randomly assigned to experimental conditions. For example, variations in positions on the multi-well plate assigned (e.g., edge positions potentially differ in temperature, air flow etc.; the delay in handling—typically from the upper left to the lower right—can introduce effects), cell passage number, confluence, or donor characteristics can introduce bias.
- *Performance bias*: can arise from differences in experimental procedures, such as variations in culture conditions (e.g., different incubators, more frequent procedures or controls on treated samples), reagent quality, or dosing methods that differ between experimental groups.
- *Detection bias*: in vitro studies can have detection bias if outcome measurements are subjective or if the person measuring outcomes knows the experimental conditions. For example, in morphological assessments, awareness of treatment conditions can influence scoring.
- *Reporting bias*: includes selective reporting of cell responses or focusing on specific outcomes that show statistical significance. For example, cell viability or cytotoxicity studies may only report positive findings.

Special considerations: In vitro studies are more vulnerable to batch effects, contamination, and reagent variability, which can influence experimental reproducibility; Blinding and standardization of protocols are essential to mitigate these biases (Henderson et al. 2013). The application of Good Cell and Tissue Culture Practices (Pamies et al. 2020, 2022, 2024) helps to limit these. They are broader in scope but overlapping with the OECD Good Laboratory Practice for in vitro methods (GIVIMP^13^).

### In silico studies

In silico studies are computational models or simulations that predict the effects of chemical or biological agents based on existing data or algorithms. While in silico studies are not subject to biases related to experimental handling, they have unique bias concerns.

Key risk of bias types

- *Selection bias*: arises from the choice of data sets or parameters used in the models. For instance, using data that is not representative of the broader population can skew results. Similarly, if training data for machine learning models are biased or incomplete, e.g., of higher quality by cherry-picking trustworthy results or over-represent certain classes of chemicals, it can result in flawed predictions.
- *Algorithmic bias*: occurs when the model itself is biased due to the algorithm’s structure or underlying assumptions. For example, overfitting a model to training data can lead to biased predictions when applied to new data.
- *Performance bias*: results from inadequate validation of the model or failure to account for variability in input parameters, which may make the model predictions unreliable or overly optimistic.
- *Reporting bias*: includes selective reporting of model outputs that show good performance metrics while ignoring models that do not perform well. Additionally, reporting can be biased by only showcasing models with high predictive accuracy without revealing validation results.

Special considerations: in silico models are sensitive to biases in the training data, parameter selection, and algorithm design. Robustness and generalizability assessments, alongside transparent reporting of model development and validation, are crucial to address these biases. There are no broadly accepted best practices for in silico methods but initial guidance exists (Tropsha 2010; Piir et al. 2018).

## The new challenge of AI-based tools and their risk of bias

Artificial intelligence (AI) and machine learning (ML) technologies are transforming toxicology by enabling new approaches to data analysis, prediction, and modeling that were previously unattainable. They represent a distinct (sub-)class of in silico models with unique features. These AI-based tools hold significant promise for accelerating research, improving predictive accuracy, and supporting decision-making in regulatory toxicology (Hartung 2023a, b; Kleinstreuer and Hartung 2024). However, as the adoption of AI increases, so do concerns about the inherent biases that these tools may introduce or perpetuate. This chapter explores the new challenges posed by AI-based tools in toxicology, focusing on the sources and types of bias specific to these technologies, their implications for research and policy, and strategies for mitigating bias to ensure the reliability and fairness of AI-driven conclusions. This represents an enormous challenge for their validation (Hartung and Kleinstreuer 2025).

### Types of bias in AI-based toxicology tools

**Fig. 4:**
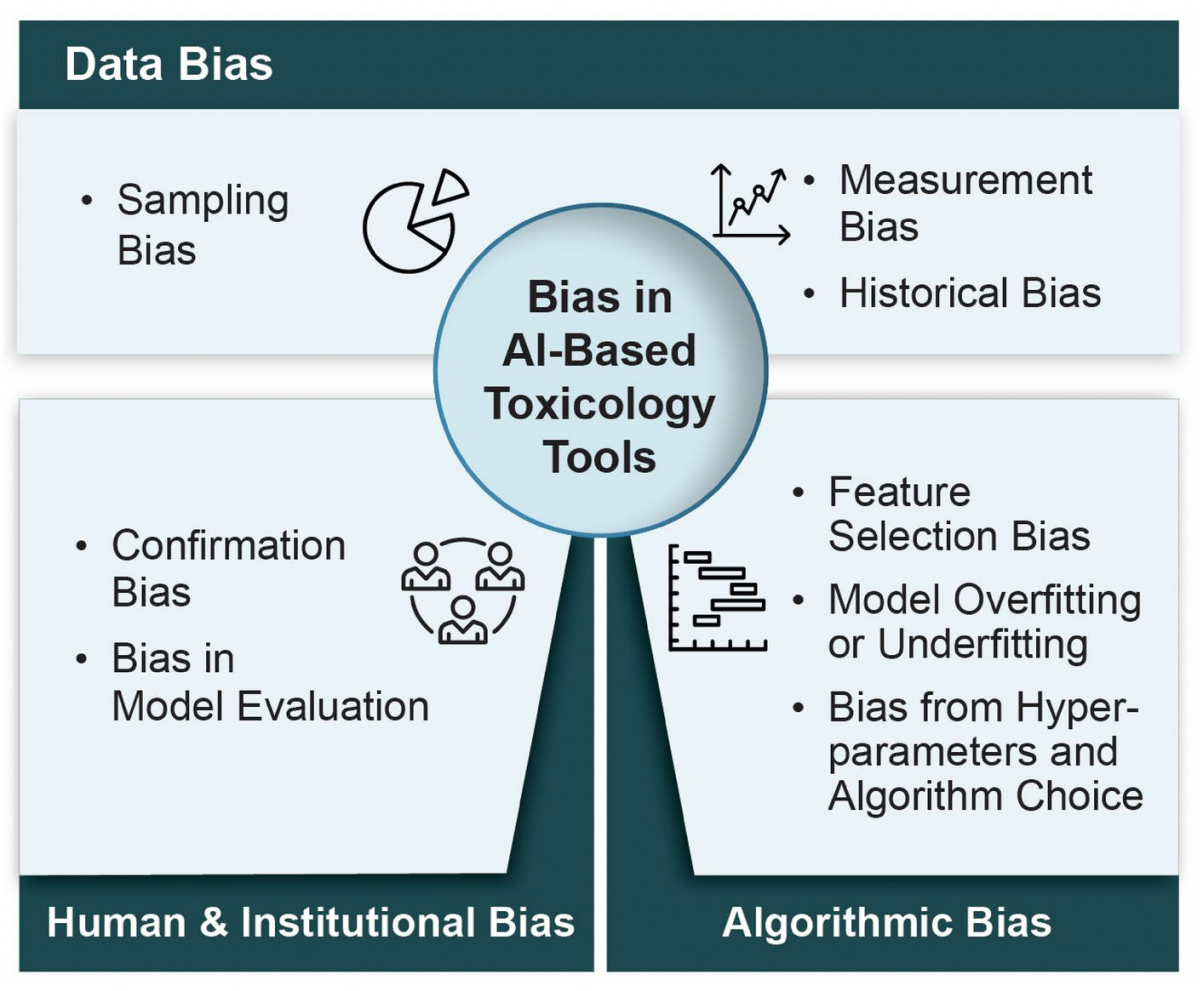
Sources of bias in AI-based toxicology tools

AI-based tools are susceptible to biases that originate from several sources, including data, algorithms, and human oversight (Zou and Schiebinger 2018; Rajkomaret al. 2018; Wiens et al. 2019; Mehrabi et al. 2021). The following are the primary types of bias that can influence AI-based toxicological models (Fig. 4):

1. Data bias, when the training data is not representative of the broader conditions of interest or measurements have not been performed the same throughout the dataset, consists of at least the following subtypes:

- *Sampling bias*: occurs when the training data used to develop AI models are not representative of the broader population or experimental scenarios. For instance, if the dataset primarily includes data from male rodents, the model’s predictions may be less accurate for female rodents or obviously for other species.
- *Measurement bias*: arises when there are inconsistencies or errors in the data collection process. This can include variations in experimental conditions, differences in data recording methods, or incomplete data, leading to systematic errors that the AI model may learn and replicate.

- *Historical bias*: results from biases present in the original data, such as overrepresentation of certain outcomes, underreporting of negative results or tendencies to under/overpredict effects based on prevalent biases in studies, which may skew the model’s predictions. Drifts and shifts over time (e.g., in animal breeding and husbandry or changed (batches of) reagents) can make historical data different; this is the key challenge, the Virtual Control Group approach (Steger-Hartmann et al. 2020; Golden et al. 2024) is facing in the starting VICT3R project.^14^

2. Algorithmic bias, in which the training or design of the AI or ML algorithms themselves introduces bias:

- *Model overfitting or underfitting*: occurs when an AI model is too closely tailored to the training data (overfitting) or when it fails to capture the underlying patterns due to excessive simplification (underfitting). This can result in biased predictions when the model is applied to new data.
- *Feature selection bias*: bias can be introduced through the selection of features used to train the model. If the selected features are not adequately validated or are inherently biased (e.g., reliance on surrogate endpoints), the model’s outputs may reflect these biases.
- *Bias from hyperparameters and algorithm choice*: the selection of model hyperparameters and algorithm types can also influence the bias of the model. Certain algorithms may be more prone to specific biases (e.g., decision trees being sensitive to imbalanced data), leading to skewed outputs.

3. Human and institutional bias is also important to consider, around how the results of the use of AI can be systematically misinterpreted or otherwise distorted:

- *Confirmation bias*: human biases can be encoded into AI models through choices made during data selection, feature engineering, or interpretation of results. For example, researchers may select specific datasets or features that align with preconceived hypotheses, inadvertently introducing confirmation bias.
- *Bias in model evaluation and validation*: the metrics used to evaluate and validate AI models (e.g., accuracy, sensitivity, and specificity) can influence how model performance is perceived. If the validation metrics are not appropriately chosen to address potential biases, the resulting models may appear robust but perform poorly in real-world applications.

### Implications of AI bias in toxicology

The implications of bias in AI-based toxicological tools are far-reaching. Biased AI models can lead to incorrect predictions of chemical toxicity, misclassification of safe or hazardous substances, and skewed risk assessments, all of which reduce the effectiveness of chemical risk management and could undermine the credibility of these tools in research and regulatory contexts. Specific concerns include:

- *Misclassification of chemical safety*: biased AI models may incorrectly classify chemicals as safe or hazardous, which could lead to inappropriate regulatory decisions. For example, underrepresentation of certain chemical classes in training data could result in false-negative predictions for toxicity, increasing the risk of public health impacts. Notably, this is no different for traditional approaches.
- *Perpetuation of existing biases*: historical biases present in toxicological data, such as gender, species, or chemical-specific biases (e.g., “overstudied” chemicals), can be perpetuated or even amplified by AI models. This is particularly problematic in fields such as developmental or reproductive toxicology, where gender differences may be pronounced, but underrepresented in historical data.
- *Challenges in reproducibility and transparency*: AI models, especially those based on complex deep learning architectures, are often considered “black boxes” due to their opacity in decision-making processes. This lack of transparency makes it challenging to identify and correct biases, impacting the reproducibility and interpretability of findings.
- *Impact on regulatory acceptance*: regulatory agencies are increasingly cautious about adopting AI models due to concerns about bias and lack of transparency. The European Food Safety Authority (EFSA), the US Environmental Protection Agency (EPA), and other agencies have emphasized the need for rigorous validation and transparency in AI tools to ensure their reliability in regulatory risk assessment.

### Mitigating bias in AI-based toxicology tools

Addressing bias in AI-based toxicology tools requires a multifaceted approach that involves improvements in data quality, algorithm design, model evaluation, and transparency. Wiens et al. (2019) while focused on healthcare, offer valuable insights into responsible AI development that can be applied to toxicology, particularly in addressing data bias and model transparency. The following strategies can help mitigate bias and enhance the reliability of AI models in toxicology:

- *Improving data quality and representativeness*: use diverse and representative datasets that encompass variations in species, sex, age, and experimental conditions to reduce sampling bias. This does not improve the data quality of the individual input data, but the ‘quality’ of the entire training data set and needs to be separated from efforts targeting improvement of the primary data quality. Implement rigorous data curation practices to ensure consistency in data collection, handling, and annotation. Address data gaps through the inclusion of new experimental data or integration with complementary data sources, such as omics data or epidemiological studies.
- *Algorithm design and feature engineering*: develop algorithms that are robust to biases in data, such as reweighting techniques, adversarial training, or debiasing layers that correct for imbalances in the training data. Employ feature selection methods that identify and exclude biased or redundant features while maintaining model interpretability. Use ensemble methods that combine predictions from multiple algorithms to reduce the impact of any single biased model.
- *Transparent reporting and validation*: implement standardized reporting guidelines, such as CONSORT-AI^15^ or TRIPOD-AI^16^ (Collins et al. 2024), to ensure transparency in model development and validation. Conduct extensive validation using independent datasets and external benchmarks to assess the generalizability and robustness of AI models. Report performance metrics disaggregated by subgroups (e.g., sex, species) to identify potential biases and performance disparities.
- *Human oversight and interpretability*: encourage human oversight in the development and application of AI models to provide context and domain expertise that can identify potential biases. Develop interpretable AI models, such as attention-based models or decision trees, which allow researchers to understand and trace the model’s decision-making process. Use explainability tools like SHAP (SHapley Additive exPlanations)^17^ or LIME (Local Interpretable Model-Agnostic Explanations)^18^ to interpret model outputs and detect bias.
- *Regulatory and policy recommendations*: regulatory agencies should establish guidelines for the validation and deployment of AI models, emphasizing bias detection, correction, and transparent reporting. Implement regulatory frameworks that require AI models to be accompanied by detailed risk of bias assessments, similar to those used for traditional toxicological studies. Promote collaboration between AI researchers, toxicologists, and regulators to develop consensus standards for bias mitigation and model evaluation.

It should be noted that none of the above measures can fully compensate for distortions in training data that result from publication and reporting bias, or from systematic shortcomings in how research is conducted, that result in issues such as over-preponderance of positive findings in the literature or systematic exaggeration or diminution of effect sizes. Identifying where such issues are in play, and ensuring this is integrated into assessments of certainty in the results of the application of AI and ML in toxicological research, is of high importance in ensuring such tools are appropriately used and given due credence. A detailed discussion of issues around this is provided by Brozek et al. (2021).

### Future directions

The future of AI in toxicology depends on our ability to recognize and mitigate biases in these models. Advances in AI techniques, such as federated learning, transfer learning, and explainable AI, offer new opportunities to address biases inherent in data and models. Moreover, interdisciplinary collaboration between AI experts, toxicologists, and regulators will be essential to developing robust, unbiased AI models that can be confidently used for chemical safety assessment and regulatory decision-making.

Ongoing research efforts should focus on:

- Developing bias-resilient AI architectures that can generalize across diverse datasets and experimental conditions.
- Establishing ethical guidelines for AI deployment in toxicology, emphasizing fairness, transparency, and accountability.
- Building comprehensive databases that integrate diverse data types (e.g., molecular, epidemiological) to improve model representativeness and reduce biases.

In conclusion, while AI holds great promise for transforming toxicology, addressing the challenges of bias is crucial to establishing stakeholders trust and realizing its full potential. By adopting best practices for data quality, model design, and transparency, and fostering interdisciplinary collaboration, the toxicological community can harness the power of AI in a responsible and unbiased manner.

## Prevalence and impact of bias in toxicological studies

Begley and Ellis (2012) discuss the implications of bias in preclinical research for drug development, highlighting the importance of addressing bias to improve translational success. Bias is a prevalent issue in toxicological research, with numerous systematic reviews indicating its potential to distort study outcomes. While not specifically about risk of bias, Moher et al. (2009) introduces reporting guidelines for systematic reviews, which are crucial for transparent assessment and reporting of bias in evidence synthesis. Macleod et al. (2014) addresses the issue of waste in biomedical research, including the impact of biases, and proposes strategies for improvement, which aligns with the section's focus on enhancing research quality. Following this, Macleod et al. (2015) provides a comprehensive analysis of risk of bias in animal studies, offering empirical evidence on the prevalence of various biases in preclinical research. Kimmelman et al. (2014) discuss how different types of bias can affect exploratory versus confirmatory research in preclinical studies, including toxicology. A systematic review by Bero et al. (2016) examined how funding sources can introduce bias in toxicological studies, which is relevant to the discussion on the prevalence and impact of bias. These findings underscore the need for systematic bias assessments and the adoption of best practices in study design and reporting.

Several studies have explored the prevalence and extent of bias in toxicological research, highlighting the significant impact it can have on study outcomes and the reliability of the evidence base. These studies have mainly focused on animal research and, to a lesser extent, in vitro studies and epidemiological research. Here are some key findings on the extent of bias in toxicological studies:

### Prevalence of bias in animal studies

Kilkenny et al. (2009) report a survey that assesses the quality of experimental design and reporting in animal studies, providing insights into the prevalence of methodological issues that can lead to bias, which led a year later to the ARRIVE reporting standards (Kilkenny et al. 2010). The cumulative evidence indicates that bias is pervasive in toxicological research, with high risks of selection bias, performance bias, and reporting bias commonly documented. The extent of bias is associated with substantial overestimation of treatment or exposure effects, highlighting the need for rigorous study design, transparent reporting, and systematic risk of bias assessment. By identifying and mitigating these biases, researchers can improve the reliability of toxicological studies and the validity of their conclusions, ultimately supporting better-informed regulatory and public health decisions.

Several (systematic) reviews have demonstrated that risk of bias is prevalent in toxicological animal research:

- *Risk of bias in animal studies:* a study by Hooijmans et al. (2014) assessed the risk of bias in animal studies using the SYRCLE Risk of Bias tool. The study found that 86% of animal studies in their sample had a high or unclear risk of selection bias due to inadequate reporting of randomization methods, and 100% of studies had high or unclear risk of performance bias because of a lack of blinding. This suggests that most animal studies are potentially compromised by high risk of bias, which could lead to overestimation of treatment effects. A study published in 2013 (Muhlhausler et al. 2013) found that only 28% of animal studies published in Cancer Research reported random allocation of animals to treatment groups, and only 2% reported allocation concealment. This suggests that a large proportion of studies may have unclear or high risks of selection bias. Another study (Brown 2006) examining clinical trials in dogs and cats found that while 87% of reports stated randomization was used to allocate animals to groups, only 11% described both randomization of the group allocation process and concealment of the allocation sequence. This indicates that even when randomization is reported, details on allocation concealment are often missing. This suggests that a significant portion of studies may not adequately report or implement these.
- *Systematic review of animal studies:* Krauth et al. (2013) conducted a systematic review of animal studies in stroke research and found that only 28% of studies used random allocation and less than 5% reported blinding of the outcome assessment. The lack of these key elements of study design increased the risk of bias, which is associated with an overestimation of treatment effects by 30% to 40%.
- *Impact of bias on effect size:* Sena et al. (2010) found that animal studies with high risk of bias reported 45% larger effect sizes compared to studies with low risk of bias in neurological research. This indicates that bias can substantially exaggerate the perceived efficacy or safety of interventions.

### Bias in in vitro studies

Bias in in vitro studies is less well-documented compared to animal studies, but there is emerging evidence of its prevalence:

- *Publication bias in in vitro studies:* a review by Henderson et al. (2013) indicated that publication bias and selective reporting are also concerns in in vitro toxicological studies. Positive findings are more likely to be published, while negative results are often underreported or omitted, which can skew the perceived safety or efficacy of compounds.

### Bias in environmental and epidemiological studies

Human observational studies and environmental health research are also susceptible to bias, with studies documenting the following:

- *Systematic review of environmental health studies:* Rooney et al. (2016) performed a systematic review of environmental health studies using the OHAT Risk of Bias tool and found that 70% of studies had a high risk of selection bias and confounding due to inadequate adjustment for key variables. This suggests that bias is prevalent and often goes unaddressed in studies informing chemical safety and public health policy.
- *Meta-analysis of publication bias:* Gurevitch et al. (2018) discuss the importance of considering bias in meta-analyses and systematic reviews, which is relevant to the section's discussion on how bias affects evidence synthesis and decision-making. A meta-analysis by Goodwin et al. (2014) on toxicological studies showed that publication bias and selective outcome reporting resulted in an overestimation of the carcinogenic potential of chemicals by up to 25%. This has significant implications for regulatory decisions and risk assessments.

## The opportunity for AI to identify risk of bias in toxicological studies

AI has the potential to revolutionize the way we assess and address risk of bias in toxicological research. Traditionally, identifying and evaluating biases in studies has been a time-consuming and subjective process that requires extensive expertise and meticulous scrutiny of research protocols, methodology, and outcomes. With the advent of AI-based tools, it is now possible to automate and enhance these processes, thereby improving the efficiency, consistency, and reliability of risk of bias assessments. This chapter explores the opportunities presented by AI in identifying risk of bias in toxicological studies, discussing current approaches, potential applications, and future directions for integrating AI into systematic review and evidence assessment workflows (Table 3).

**Table 3:**
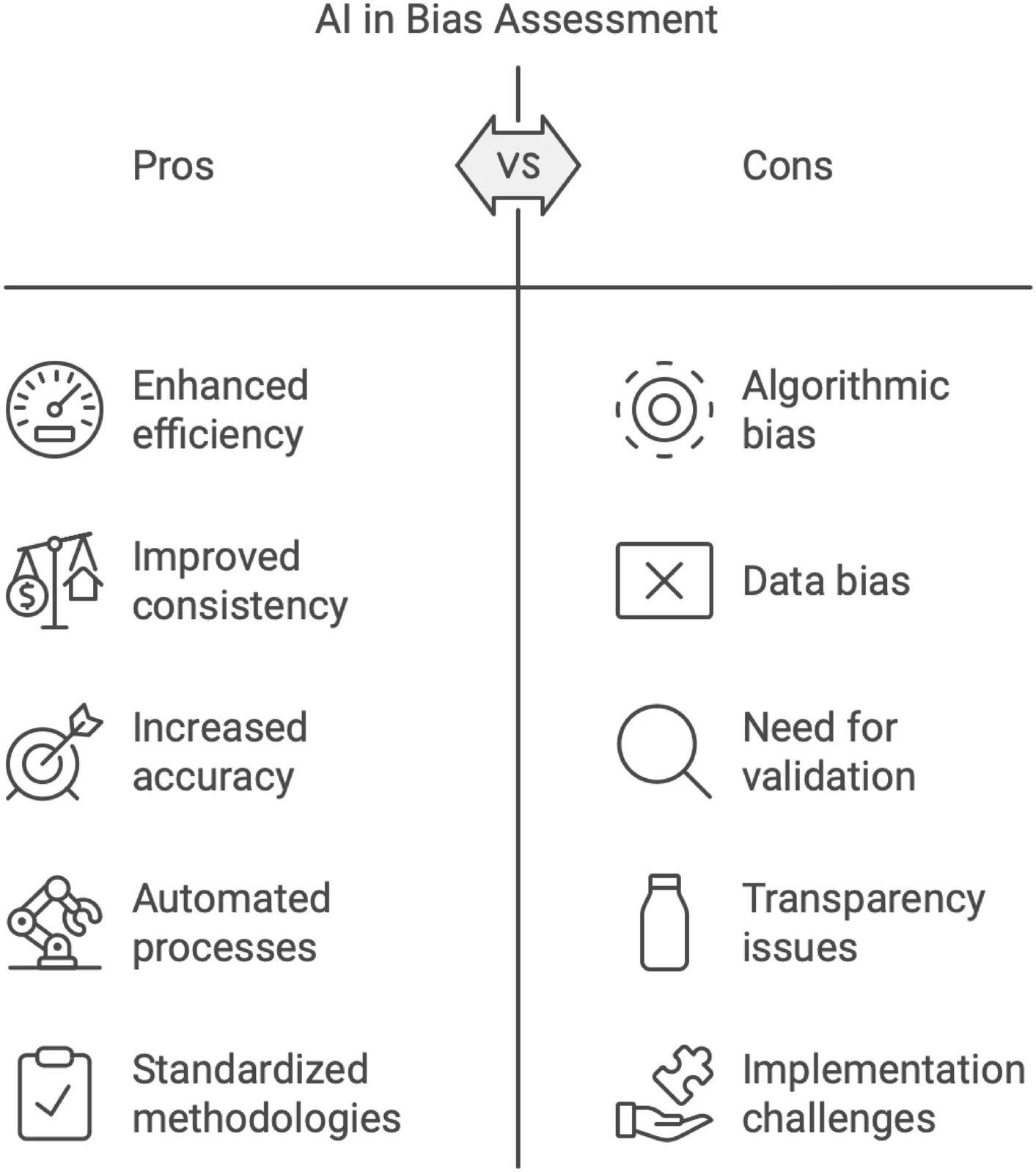
Pros and cons of AI-facilitated risk-of-bias assessment

Done with Napkins.ai

### Two paradigms for AI in bias assessment

The application of AI in bias assessment can follow two distinct paradigms, each with its own advantages and challenges. The first approach focuses on automating traditional risk of bias assessment by mimicking human reviewer processes. This method employs natural language processing to extract methodological features and applies predefined assessment criteria. While this approach offers immediate practical benefits, it remains confined by existing frameworks and depends heavily on reporting quality, potentially perpetuating current limitations in bias assessment.

The second paradigm represents a novel, computer-enabled approach to bias detection. This method leverages unique computational capabilities to identify patterns in study outcomes and discover new indicators of bias. It offers several advantages over traditional approaches, including the ability to detect previously unknown sources of bias, process significantly larger datasets, and provide more objective measurements of actual bias. However, this approach also faces important challenges, including the need for thorough validation, the potential introduction of new biases, and the requirement for transparent methodology.

These dual approaches highlight the critical need for improved methods reporting in scientific literature. This starts with Open Science,^19^ i.e., open access publishing and FAIR^20^ (**F**indability, **A**ccessibility, **I**nteroperability, and **R**euse of digital assets) data access. The Promoting Reusable and Open Methods and Protocols (PRO-MaP) recommendations (European Commission 2024) begin from the premise that scientific journals should strive to publish articles that are fully reproducible and reusable. Future research reports should incorporate structured, machine-readable methods sections, standardized reporting formats, and complete methodological documentation that can integrate seamlessly with automated assessment tools.^21^ This evolution in scientific reporting would support both traditional assessment automation and the development of novel computational methods for bias detection.

The implementation of these approaches requires careful consideration of both their potential benefits and limitations, along with a commitment to maintaining transparency and scientific rigor throughout the assessment process. As these methods continue to evolve, they will likely play an increasingly important role in ensuring the quality and reliability of scientific research.

### Potential applications of AI in identifying risk of bias

AI can be applied at various stages of research evaluation, from initial study screening to detailed assessment of study quality. The following are key areas where AI can be leveraged to identify and mitigate risk of bias:

- *Automated screening and data extraction*: one of the most labor-intensive tasks in systematic reviews and meta-analyses is the screening of studies for inclusion and extraction of relevant data. AI-based text mining and natural language processing (NLP) techniques can automate this process by scanning large volumes of literature and identifying studies that meet predefined inclusion criteria. For example, AI tools can be trained to recognize and extract information related to randomization procedures, blinding, sample size, and outcome measures, which are crucial for assessing risk of bias. This automation not only accelerates and scales data retrieval but reduces human error and inter-rater variability, ensuring that the assessment is more consistent and less prone to subjective interpretation. Rerunning manual systematic reviews with automated tools can identify missed or wrongly classified publications.
- *Identification of reporting bias*: AI algorithms can be developed to detect selective reporting and publication bias by comparing reported outcomes to protocol documents or analyzing trends in the reporting of statistically significant results. The techniques such as text mining can identify discrepancies between the study’s registered protocol (e.g., ClinicalTrials.gov^22^) and the published report, highlighting unreported or altered outcomes. AI can also be used to analyze large datasets to detect patterns indicative of publication bias, such as the overrepresentation of positive findings. Machine learning models can identify trends that suggest underreporting of negative or null results, enabling researchers to systematically flag potential reporting biases.
- *Detection of statistical bias and inconsistencies*: AI can perform statistical analyses to identify anomalies or patterns that indicate biases in study results. AI excels at pattern recognition supervised and unsupervised beyond scaling and automation of traditional statistical approaches. For example, meta-analysis tools equipped with AI can detect inconsistencies in effect sizes that suggest selective inclusion of data points or deviations from expected distributions, which may indicate reporting or performance bias. Advanced AI models can also perform sensitivity analyses to evaluate the robustness of study findings under different assumptions, providing insights into the potential impact of biases on study outcomes.
- *Assessment of study design and methodology*: AI can assist in the evaluation of study design features, i.e., assess reporting quality, by analyzing how closely the methodologies adhere to standardized guidelines such as ARRIVE (Animal Research: Reporting of In Vivo Experiments) or OECD (Organization for Economic Co-operation and Development) principles.^23^ AI can beyond this improve today risk of bias assessment, e.g., machine learning models can be trained to recognize language indicative of high-risk practices, such as lack of randomization or insufficient blinding. NLP tools can parse study protocols and identify elements that are critical for assessing risk of bias, such as random sequence generation, allocation concealment, and blinding procedures. This automated evaluation can highlight studies that may require further scrutiny or exclusion due to potential biases.
- *Predictive modeling for bias identification*: AI can be leveraged to build predictive models that estimate the likelihood of bias in a given study based on historical data. By training on datasets annotated for bias (e.g., Cochrane reviews or SYRCLE risk of bias assessments), AI models can predict risk of bias scores for new studies, prioritizing them for detailed manual review. Such predictive models can be fine-tuned to account for different types of toxicological studies (in vivo, in vitro, and in silico) and incorporate contextual information such as study design, sample size, and outcome measures, enabling a more tailored and accurate assessment of bias risk.

**Fig. 5:**
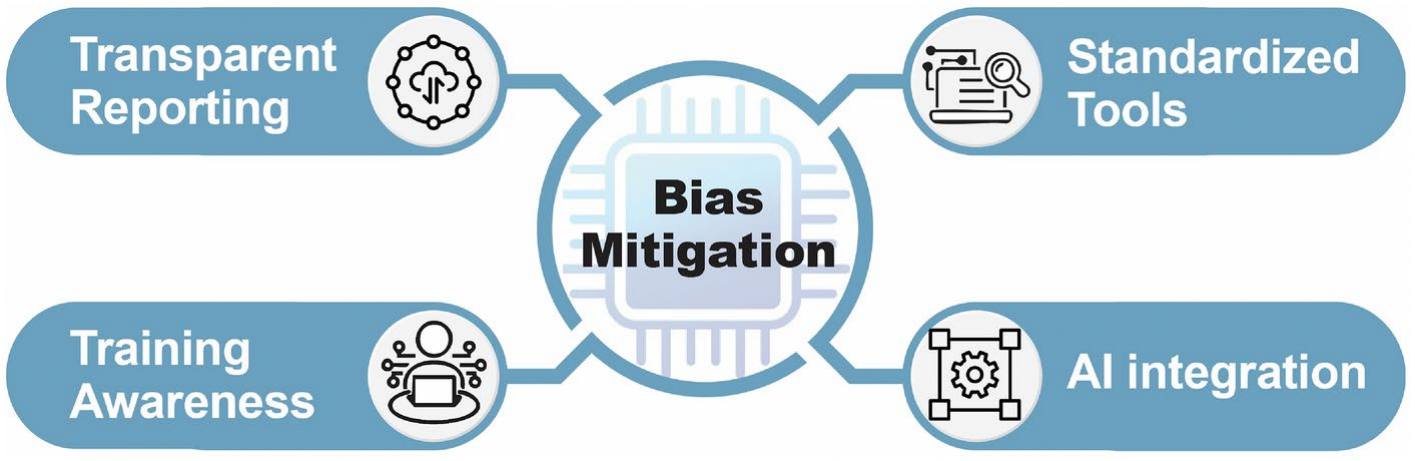
Principle approaches in bias mitigation. This illustrates how bias (selection, reporting, performance) can be mitigated: transparent reporting (e.g., standardized reporting formats), increased awareness of users, standardization of research tools employed, and increasingly AI-based tools for bias detection

Opportunities for AI to mitigate risk of bias in research are illustrated in Fig. 5.

### Case studies: AI in action for bias detection

Bannach-Brown et al. (2019) demonstrate the application of machine learning to screen studies for inclusion in systematic reviews of animal studies, which is relevant to toxicology. More recent work by the US National Toxicology Program has provided proof-of-concept for use of NLP in data extraction in literature review (Walker et al. 2022). Several pioneering applications have demonstrated the feasibility and efficacy of AI in identifying risk of bias in scientific research:

- *RobotReviewer*: RobotReviewer^24^ (Marshall et al. 2016) is an AI-based tool that automatically extracts and assesses risk of bias from clinical trial reports. Using NLP and machine learning, RobotReviewer can evaluate study characteristics such as random sequence generation, allocation concealment, and blinding. While initially developed for clinical trials, the underlying technology can be adapted for toxicological studies, offering a template for automated bias assessment. Application in Toxicology: The methodology used by RobotReviewer can be applied to extract key methodological details from toxicological studies, highlighting studies with insufficient randomization or unclear blinding.
- *Risk of Bias (RoB) Suite*: The Risk of Bias (RoB) Suite^25^ is a collection of AI tools developed to automate the extraction and assessment of risk of bias in preclinical research. RoB Suite uses Natural Language Processing (NLP) to identify and evaluate risk of bias domains in study reports, reducing the time required for manual review. It has been successfully applied to large datasets of animal studies (Wang et al. 2022), demonstrating the potential of AI in preclinical research evaluation. Application in Toxicology: RoB Suite can be adapted for systematic reviews of animal toxicology studies, identifying potential biases related to sample allocation, blinding, and reporting practices.
- *PubMed mining and trend analysis*: AI-based text mining and trend analysis have been used to identify potential reporting biases in the scientific literature (Peters et al. 2006). For example, text mining algorithms can analyze publication trends over time to identify shifts in the reporting of outcomes, which may indicate the presence of publication bias. Such tools can provide an early warning system for biases in the toxicological evidence base. Application in toxicology: trend analysis can be used to monitor the publication patterns of toxicological studies, flagging potential cases of selective reporting or underreporting of adverse effects.

In general, AI is most capable of identifying bias,^26^,^27^ (Soboczenski et al. 2019). For example, AI Fairness 360 (AIF360)^28^ is IBM's extensible open-source toolkit providing algorithms and metrics to detect, understand, and mitigate unwanted algorithmic biases in machine learning models.

AI-facilitated systematic review SWIFT-Review (Howard et al. 2016) is such a text mining tool that can be used to prioritize studies for screening in systematic reviews, including those in toxicology. While not specifically about AI, Lau et al. in their seminal paper (1998) discuss the importance of systematic reviews and meta-analyses in synthesizing evidence, which is relevant to the discussion of AI-assisted bias detection. Borah et al. (2017) quantify the time and resources required for systematic reviews, highlighting the potential benefits of AI-assisted approaches in toxicology.

The spin-off Insilica LLC of our own research, released SysRev.com^29^ in 2019. It is a web-based platform designed to facilitate collaborative data extraction and systematic evidence reviews (SERs).^30^ Key features and aspects of SysRev include: (1) enabling users to create data curation projects called "sysrevs", (2) allowing uploading documents, defining review tasks, recruiting reviewers, and performing review tasks, (3) supporting a wide range of review-based tasks, including extracting large datasets for machine learning, conducting various types of literature reviews (narrative, scoping, systematic), and creating evidence gap maps. Users can search for and store documents from various sources and carry out collaborative reviews. It combines human and machine learning algorithms for data extraction and insight generation applying the FAIR Principles (Bozada et al. 2021). Free accounts can create unlimited publicly accessible projects; private Projects from paid accounts can create private projects with restricted access. SysRev aims to address issues of siloed, unstructured, and inaccessible data while promoting evidence-based decision-making and integrating artificial intelligence with human reasoning across disciplines.

### Future directions for AI in risk of bias identification

**Fig. 6:**
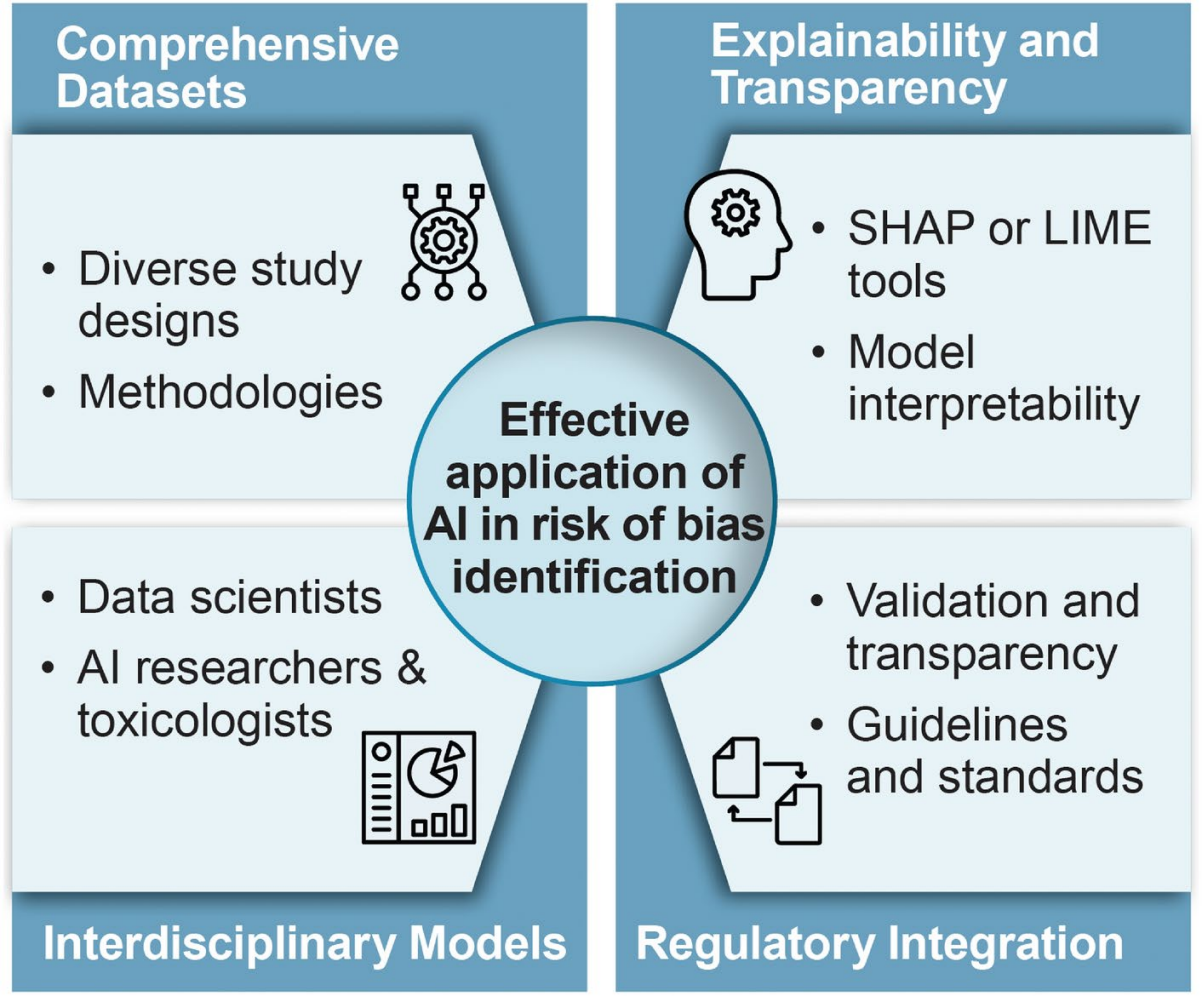
AI for risk-of-bias identification. AI applications (e.g., screening, data extraction, anomaly detection) with types of bias (selection, reporting, performance). This illustrates how AI can be strategically deployed to address multiple biases in research workflows

The application of AI in risk of bias identification is still in its early stages, but it is poised to become a transformative tool in toxicological research. Future developments should focus on (Fig. 6):

- *Creating comprehensive, annotated datasets*: building comprehensive datasets of toxicological studies annotated for risk of bias domains will be critical for training robust AI models. These datasets should include diverse study designs and methodologies to ensure generalizability across different types of toxicological research. Bender and Friedman (2018) discuss the importance of comprehensive, annotated datasets and transparency in AI model development. Boughorbel et al. (2017) discuss techniques for handling imbalanced datasets, which is relevant to creating comprehensive, annotated datasets for training AI models in risk of bias assessment.
- *Developing interdisciplinary AI models*: collaborative efforts between AI researchers, toxicologists, and data scientists are needed to develop interdisciplinary models that can integrate complex data types (e.g., experimental data, ~omics data) and account for domain-specific biases.
- *Incorporating explainability and transparency*: AI models used for bias identification should be transparent and interpretable, allowing researchers to understand how decisions are made and to validate the findings. Explainability tools, such as SHAP or LIME (Ribeiro et al. 2016), should be incorporated to provide insights into the model’s decision-making process. As an example, Lundberg and Lee (2017) introduced SHAP (SHapley Additive exPlanations), as a tool for incorporating explainability and transparency in AI models for bias detection.
- *Regulatory acceptance and integration*: engaging with regulatory agencies to develop guidelines and standards for the use of AI in risk of bias assessment will be essential for widespread adoption. Regulatory frameworks should establish criteria for the validation and transparency of AI tools, ensuring their credibility and reliability.
- *Leveraging federated learning and privacy-preserving AI*: federated learning (Yang et al. 2019), where AI models are trained across multiple datasets without data sharing, can be used to develop robust models for bias detection without compromising data privacy. This approach could facilitate the development of AI tools that draw on proprietary or sensitive data without requiring data centralization.

In conclusion, AI-based tools offer unprecedented opportunities to improve the identification and mitigation of risk of bias in toxicological studies. By automating labor-intensive tasks, enhancing consistency in evaluations, and providing new insights into patterns of bias, AI can help address some of the long-standing challenges in toxicological research. The successful integration of AI into systematic review workflows will require interdisciplinary collaboration, robust validation, and adherence to principles of transparency and fairness. As AI continues to evolve, it will play an increasingly critical role in ensuring the reliability and validity of toxicological evidence, supporting better-informed regulatory decisions and advancing the field of evidence-based toxicology.

## Conclusions and the way forward

Risk of bias assessment has emerged as a critical component in the evaluation of study quality and the overall integrity of scientific evidence in toxicology. Over the years, the field of toxicology has grappled with issues related to reproducibility, inconsistencies and variability in study outcomes, and conflicting evidence—all of which can partly be attributed to various biases inherent in study design, conduct, analysis, and reporting. Here, we highlight the need for systematic risk of bias assessment in toxicological studies to ensure that the evidence base used for regulatory decision-making and public health policies is robust and reliable (Fig. 5).

The primary sources of bias identified in toxicological research include selection bias, performance bias, detection bias, attrition bias, and reporting bias. Each of these biases can significantly impact study outcomes, potentially leading to an overestimation or underestimation of effects, which ultimately affects the credibility of the findings. Tools such as the SYRCLE Risk of Bias Tool, the OHAT Risk of Bias Tool, and the Navigation Guide have been developed or adapted to address these concerns, providing structured frameworks for evaluating biases in different study designs, including animal experiments, in vitro studies, and human observational research.

The use of AI for identifying and mitigating risk of bias is a recent and promising development in toxicological research. AI-based tools can automatically screen studies for indicators of bias, detect inconsistencies, and even predict the likelihood of bias based on historical data. These tools offer unprecedented opportunities to enhance the efficiency and accuracy of risk of bias assessments, reduce human subjectivity, and provide new insights into the sources of bias. However, the integration of AI introduces new challenges, such as algorithmic and data biases, which must be addressed to ensure the reliability and fairness of AI-driven conclusions.

Systematic reviews and meta-analyses in toxicology have shown that bias is pervasive, with high risks reported across various study designs. For instance, the large majority of animal studies show high or unclear risks of selection bias due to inadequate randomization or concealment of group allocation. Similarly, performance and detection biases are prevalent due to a lack of blinding and differential treatment or assessment across groups. In vitro studies are particularly susceptible to biases related to cell line variability, batch effects, and selective reporting. In silico studies, while not subject to experimental biases, face issues with data selection, algorithmic bias, and validation practices.

Addressing these biases is critical not only for improving the quality of individual studies but also for enhancing the consistency and coherence of the overall evidence base. The researchers, journal editors, and regulatory bodies must prioritize risk of bias assessment and implement strategies to mitigate these biases at the design, conduct, and reporting stages of research.

1. *Adoption and standardization of risk of bias tools:* one of the major challenges in toxicological research is the lack of standardized and widely adopted tools for assessing risk of bias across different study types. To address this, researchers should consider adopting or adapting existing tools such as the OHAT tool for environmental health studies. Incorporating these tools into routine practice will facilitate more consistent and transparent reporting of risk of bias and allow for better comparison and synthesis of study results (Frampton et al. 2022; Whaley et al. 2024). Noteworthy, the Evidence-based Toxicology Collaboration (EBTC) is discussing how to address this,^31^ in particular in the Evidence Synthesis Working Group.
2. *Integration of AI-based risk of bias assessment:* AI-based tools can up-scale traditional risk of bias assessments by automating labor-intensive tasks and providing more consistent evaluations. These tools can be used to identify reporting biases, and detect statistical anomalies indicative of bias. By leveraging AI in risk of bias assessment, researchers can enhance the reliability of their findings and focus manual reviews on studies flagged by the AI.
3. *Development of novel tools for emerging study types:* with the growing use of in vitro systems, high-throughput screening methods, and in silico models, there is a pressing need to develop or adapt risk of bias tools that are suited for these newer methodologies. Existing tools may not fully capture the nuances of these study designs, and novel tools that consider specific sources of bias (e.g., algorithmic bias in computational models) will enhance the reliability of these approaches. For example, the authors are part of the INVITES-IN (IN VITro Experimental Studies INternal validity) project within the European Partnership for the Assessment of Risks from Chemicals (PARC). PARC aims to develop next generation chemical risk assessment to advance research, share knowledge and improve skills, protecting human health and the environment. PARC is a large-scale research and innovation 7-year (2022–2029), which involves 200 partners across 28 countries, including national agencies, research organizations, and EU bodies with a total budget of 400 million euros (50% by the EU and 50% by Member States). The INVITES-IN tool for evaluation of the internal validity of in vitro studies is needed to include the data as evidence in systematic reviews and chemical risk assessments. The tool will be designed specifically to be applied to cell culture studies, including, but not restricted to, studies meeting the new approach methodology (NAM) definition (Svendsen et al. 2023, 2024; Vist et al. 2024; Mathisen et al. 2024).
4. *Mitigating bias in AI-based tools:* AI-based tools themselves are susceptible to biases stemming from data selection, feature engineering, and algorithm design. It is essential to develop robust, transparent, and interpretable AI models that are validated using diverse datasets. Implementing explainability tools such as SHAP (SHapley Additive exPlanations) (Mangalathu et al. 2020) or LIME (Local Interpretable Model-agnostic Explanations) (Zafar and Khan 2021) can help detect and address biases within AI models.
5. *Training and capacity building:* researchers, reviewers, and regulatory bodies need training on how to use risk of bias tools and AI-based technologies effectively and consistently. Workshops, webinars, and dedicated training programs on risk of bias assessment and AI applications should be developed to build capacity in the research community. Such initiatives will ensure that risk of bias assessments are not only accurate but also reproducible across different assessors and contexts.
6. *Transparency and open science practices:* embracing transparency and open science practices, such as sharing raw data, protocols, and analysis codes, can help mitigate some sources of bias, particularly reporting and publication biases. Platforms for pre-registration of studies, like OSF (Open Science Framework),^32^ and data repositories for sharing experimental data will promote openness and allow for independent verification and re-analysis of findings.
7. *Policy and guideline implementation:* regulatory bodies and funding agencies should incorporate risk of bias assessments into their guidelines for research funding and regulatory submissions. This includes registration/publication of study protocols before study conduct, setting minimum standards for study quality and requiring detailed risk of bias evaluations as part of systematic reviews used in regulatory risk assessments. By doing so, these agencies can drive improvements in the quality of research used to inform policy decisions.
8. *Leveraging technology for bias detection:* advances in natural language processing (NLP) and machine learning offer new opportunities for automated risk of bias detection and evaluation. Developing algorithms that can screen for common indicators of bias in study reports and integrating these tools into systematic review workflows could streamline and enhance the rigor of risk of bias assessments.
9. *Creating a culture of bias awareness:* finally, fostering a research culture that acknowledges and actively addresses bias is crucial for advancing evidence-based toxicology. This involves not only understanding the different types of biases but also promoting a mindset that seeks to minimize bias through meticulous study design, conduct, and reporting practices.

In summary, the future of toxicological research depends on our ability to produce high-quality, reliable evidence free from bias. Through the adoption of standardized risk of bias tools, integration of AI-based solutions, and embracing open science principles, we can enhance the internal validity of toxicological studies. These efforts will ultimately support better-informed regulatory decisions, improved public health outcomes, and greater trust in the scientific process. As the field continues to evolve, ongoing efforts to refine and develop new methodologies for risk of bias assessment will be essential in ensuring that toxicological research meets the highest standards of scientific rigor and transparency.

## Data Availability

No novel data was produced for this manuscript.

## Footnotes

1 SEVCO Scientific Evidence Code System: https://fevir.net/resources/CodeSystem/27270#SEVCO:00001.

2 https://www.gradeworkinggroup.org/.

3 https://training.cochrane.org/handbook.

4 https://methods.cochrane.org/bias/resources/rob-2-revised-cochrane-risk-bias-tool-randomized-trials.

5 https://ntp.niehs.nih.gov/whatwestudy/assessments/noncancer/riskbias.

6 https://ntp.niehs.nih.gov/sites/default/files/ntp/ohat/pubs/handbookmarch2019_508.pdf.

7 https://joint-research-centre.ec.europa.eu/scientific-tools-and-databases/toxrtool-toxicological-data-reliability-assessment-tool_en.

8 http://www.test.scirap.org.

9 https://prhe.ucsf.edu/navigation-guide.

10 https://www.oecd.org/en/topics/sub-issues/testing-of-chemicals/good-laboratory-practice-and-compliance-monitoring.html.

11 https://www.aalas.org.

12 https://felasa.eu.

13 https://www.oecd.org/en/publications/guidance-document-on-good-in-vitro-method-practices-givimp_9789264304796-en.html.

14 https://www.vict3r.eu.

15 https://www.clinical-trials.ai/consort

16 https://osf.io/zyacb/.

17 https://shap.readthedocs.io/en/latest/.

18 https://c3.ai/glossary/data-science/lime-local-interpretable-model-agnostic-explanations/.

19 https://www.frontiersfoundation.org/open-science-charter.

20 https://www.go-fair.org/fair-principles/.

21 https://insight7.io/ai-tools-for-research-bias-detection/.

22 https://clinicaltrials.gov.

23 https://www.oecd.org/en/topics/sub-issues/assessment-of-chemicals/harmonised-templates.html.

24 https://www.robotreviewer.net.

25 https://methods.cochrane.org/risk-bias-2.

26 https://www.turingpost.com/p/ai-fairness-tools.

27 https://insight7.io/ai-tools-for-research-bias-detection/.

28 https://aif360.res.ibm.com.

29 https://www.sysrev.com.

30 https://www.perplexity.ai/search/find-refrences-for-bias-is-oft-i09XYItFRLi66Zib4UyVVQ.

31 https://www.ebtox.org.

32 https://osf.io.
